# Supplementary figures and images for: Establishment of two pathomic-based machine learning models to predict CLCA1 expression in colon adenocarcinoma
Source: PLoS One. 2025 Jul 21;20(7):e0328220. doi: 10.1371/journal.pone.0328220 (PMC12279125; doi:10.1371/journal.pone.0328220)

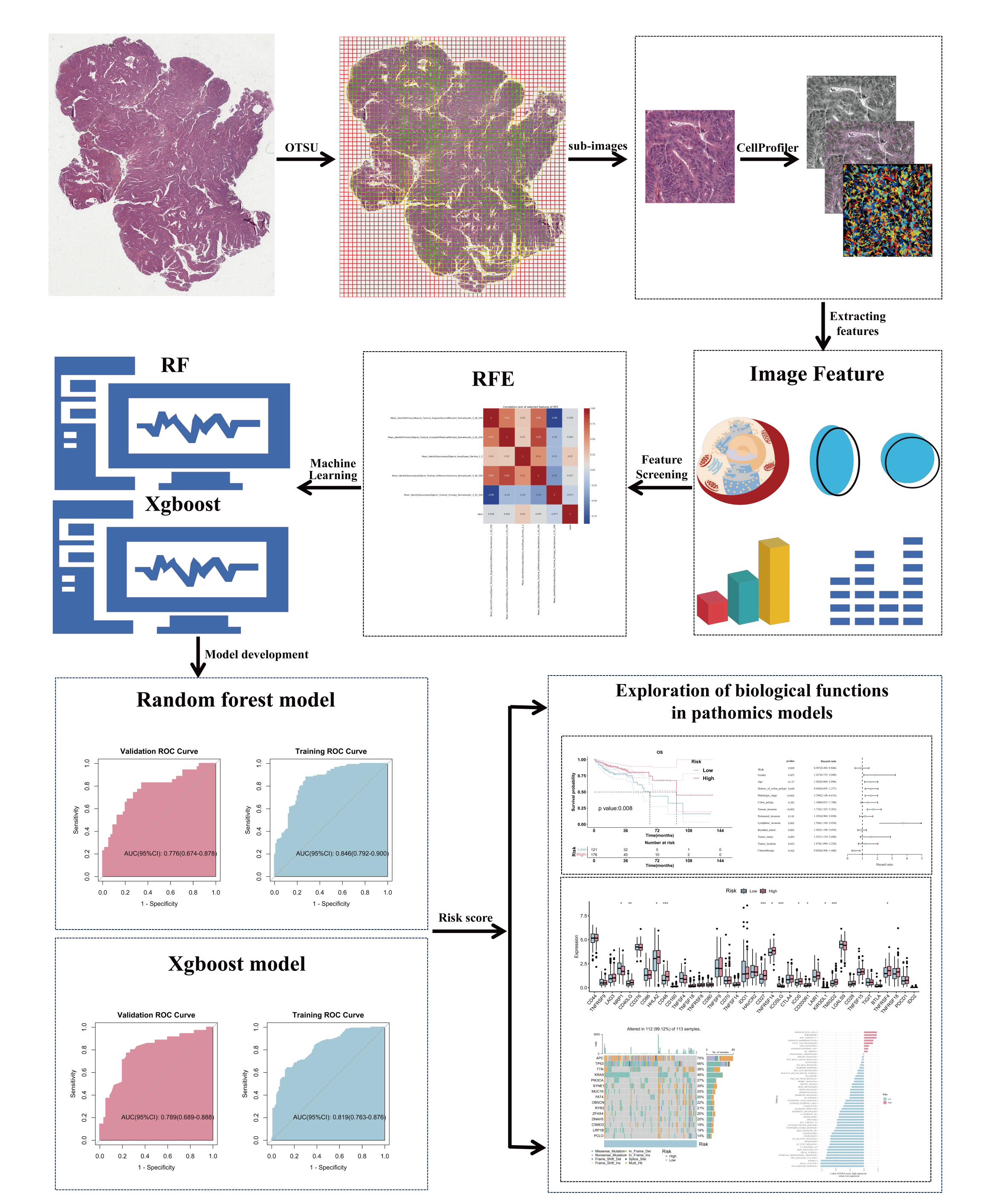

Supplement: S1 Fig — (TIF) [file pone.0328220.s008.tif]
