# Supplementary material for: Establishment of two pathomic-based machine learning models to predict CLCA1 expression in colon adenocarcinoma
Source: PLoS One. 2025 Jul 21;20(7):e0328220. doi: 10.1371/journal.pone.0328220 (PMC12279125; doi:10.1371/journal.pone.0328220)
Supplement: Attachment — Submitted filename: Response to Reviewers.docx [file pone.0328220.s009.docx]

Dear Editor,

We would like to express our sincere gratitude to you and the reviewers for the insightful comments and valuable suggestions provided during the review of our manuscript titled " Establishment of two pathomic-based machine learning models to predict CLCA1 expression in colon adenocarcinoma."We have carefully considered all the feedback and have made revisions to improve the quality of our manuscript. Below, we provide a detailed response to reviewers' comments and outline the specific changes made to the manuscript.

**Response to Reviewer 1:**

**Comment 1:** It is recommended to include a flowchart of the study to visually present the research design, data processing, and analysis steps, enhancing the readability and logical clarity of the study.

**Response 1:** Thank you for your suggestion. We developed a flowchart encompassing pathomics establishment and prognostic analysis to clarify the research process.

**Comment 2:** The authors state " CLCA1 expression was lower in cancer tissues of various tumors, including COAD, according to the differential analysis results (*p* < 0.001)". However, *p* < 0.001 implies that the authors have used only nominal p-values without multiple hypothesis correction. The authors should use FDR or similar to control false-positive rate.

**Response 2:** Thank you for pointing this out. We applied the Benjamini-Hochberg adjustment to our P values to enhance the reliability of our statistical results.

**Comment 3:** Please provide the full definition of abbreviations the first time they are used, and include the abbreviation in parentheses.

**Response 3:** We appreciate your reminder. We reviewed the entire document and supplemented any incomplete abbreviations.

**Comment 4:** Some previous studies have utilized similar bioinformatics and statistical methods (PMID: 39702250; PMID: 37437243; PMID: 36213111). While referencing these studies may reduce the novelty of the work to some extent, I believe it would greatly benefit the readers by providing a more comprehensive understanding of the context of the current study.

**Response 4:** Thank you for your suggestions! The references you suggested are beneficial to us. We have read through all the references and learned a great deal. They played a key role in improving the quality of our article. Therefore, we have cited them in the revised manuscript. Thanks once again.

**Comment 5:** I recommend that the manuscript undergo professional language editing and proofreading to improve clarity.

**Response 5:** We appreciate your reminder. We have carefully proofread the entire manuscript and corrected all typographic and grammatical errors.

Please review these changes.

We hope these modifications meet the reviewer's requirements, and we thank you again for your valuable feedback.

Best regards,

Yours sincerely,

Yang Cao

Yiwu central hospital, Jinhua, Zhejiang, China

Pathology Department. Yiwu, 322000, China

Email: [1814218039@qq.com](mailto:1814218039@qq.com)
